# COVID-19 Vaccination-Associated Myocarditis: A Literature Review

**DOI:** 10.7759/cureus.32022

**Published:** 2022-11-29

**Authors:** Vijayalakshmi Mannan, Tejasvi Kashyap, Aqsa Akram, Muhammad Sanusi, Asma A Khan, Elina S Momin, Muhammad Ahad Pervaiz, Abeer O Elshaikh

**Affiliations:** 1 Medicine, California Institute of Behavioral Neurosciences & Psychology, Fairfield, USA; 2 General Practice, California Institute of Behavioral Neurosciences & Psychology, Fairfield, USA; 3 Internal Medicine, Dallah Hospital, Riyadh, SAU; 4 Internal Medicine, California Institute of Behavioral Neurosciences & Psychology, Fairfield, USA; 5 Internal Medicine, Shenyang Medical College, Shenyang, CHN; 6 Urology, California Institute of Behavioral Neurosciences & Psychology, Fairfield, USA; 7 Internal Medicine/Family Medicine, California Institute of Behavioral Neurosciences & Psychology, Fairfield, USA

**Keywords:** covid-19 vaccination, covid-19 vaccine complication, covid vaccine-induced myocarditis, coronavirus disease, coronavirus vaccine, sars-cov-2, covid and myocarditis, covid-19 vaccine, covid-19

## Abstract

Myocarditis is defined as a non-ischemic inflammation of the middle layer of the heart. It ensues changes that can lead to acute heart failure, dilated cardiomyopathy, and sudden death. Myocarditis is caused by several infectious and non-infectious agents. Vaccines are also known to cause myocarditis. The use of the coronavirus disease (COVID-19) vaccination was started to combat the severity of the COVID-19 infection and reduce the mortality and morbidity associated with it. The vaccination, however, caused side effects like myocarditis, among others. In order to investigate the association between the COVID-19 vaccination and myocarditis in adults and adolescents, we conducted a literature review by searching three databases: Google Scholar, PubMed, and ScienceDirect. From the published literature, we found that, though it is rare, the various vaccinations available can cause symptoms of myocarditis as a side effect more commonly in patients who have received both doses of a particular vaccine and that there are significant changes in cardiac magnetic resonance imaging (CMRI) and other biochemical markers, with young males being more commonly affected. Further prospective trial-based studies are required to establish a concrete relationship between myocarditis and the COVID-19 vaccine.

## Introduction and background

Myocarditis is defined as the inflammation of the middle layer of the heart, causing clinical, histological, and immunological changes [1], which can lead to acute heart failure, dilated cardiomyopathy, and sudden death [2]. Causes of myocarditis include infectious and non-infectious agents. Infectious causes include viral agents (Adenovirus, Enterovirus, Herpes virus, HIV, and Influenza virus), bacterial agents (like Mycoplasma Pneumonia, Streptococcal species, and Treponema Pallidum), fungal agents (Aspergillus species, Candida species, and Coccidioides species), and parasitic agents [2]. Non-infectious causes include toxins, medications, and immunological syndromes like eosinophilic granulomatosis with polyangiitis, sarcoidosis, and inflammatory bowel disease, among others [2]. Over the past few years, viral etiologies, including coronavirus, have been studied and researched more than other causes [2].

Severe acute respiratory syndrome coronavirus 2 (SARS-CoV-2) belongs to the Coronaviridae family and is a positive-sense single-stranded ribonucleic acid-containing virus, responsible for causing coronavirus disease of 2019 (COVID-19), fulminant pneumonia, and acute respiratory distress syndrome (ARDS) [3]. The virus attacks the cells containing the angiotensin-converting enzyme-2 (ACE2) receptors, which are predominantly present in the lungs [3]. Besides the lungs, the intestine, kidney, heart, and blood vessels also contain ACE2 receptors, making COVID-19 a multi-system infection that demanded the immediate manufacture and use of vaccination against the virus [3].

In December 2021, in the United States of America, the Food and Drug Administration granted emergency use authorization for two vaccines, BNT162b2 by Pfizer-BioNTech and Moderna vaccine, for people over 16 years of age [4], following which their use was initiated in other countries as well. These vaccines entailed some side effects, which ranged from mild and transient to severe and life-threatening. Myocarditis in relation to vaccination has been previously described for vaccines, most notably the smallpox vaccine, along with vaccines for influenza and hepatitis B [5-7]. The Centers for Disease Control and Prevention (CDC) released statements regarding myocarditis being a potential side effect of the COVID-19 vaccine, commonly seen in young adult males [5].

The primary goal of this literature review is to examine the connection between COVID-19 vaccinations and myocarditis. We gathered 24 scholarly articles from Google Scholar, PubMed, and ScienceDirect databases based on relevance to this topic. We included full-text peer-reviewed scholarly articles from 2020 to 2022 with adolescents and adults as participants. We included randomized controlled trials, systematic reviews, meta-analyses, cohort studies, and traditional reviews. We excluded articles in languages other than English, studies done on pregnant women and animals, and studies before 2020.

## Review

Myocarditis may manifest as acute cardiac failure, cardiogenic shock, or sudden cardiac death [1,8]. Sometimes there may be ventricular arrhythmias; severe ventricular dysfunction may also occur, in which case assessment of left ventricular function has proven to be useful [2]. In most cases, however, myocarditis may be mild and self-limiting and may not warrant serious interventions [6].

Clinical features and laboratory findings

Like any other vaccine, COVID-19 vaccines frequently lead to minor side effects like pain, redness, and swelling at the injection site and more systematic effects like fever with chills, fatigue, headache, and muscle pain [8,9]. Signs of myocarditis include tachypnea, tachycardia, murmur, gallop, diminished pulses, hypotension, edema, and signs of low cardiac output [6]. In patients diagnosed with myocarditis, the symptoms ranged from mild and transient to eventually causing acute heart failure and sudden death [8,9]. The symptoms following the second dose were more severe than those following the first. In a study by Witberg et al., 81% of patients had symptoms after the second dose [10].

In a retrospective cohort study by Mevorach et al., among 9,289,765 Israeli residents who were administered the Pfizer-BioNTech COVID-19 vaccine, a total of 136 patients were reported to have definite or probable myocarditis, where 19 patients presented after the first dose and 117 presented after the second dose of the vaccine [11]. They presented with mild to moderate symptoms and had elevations in inflammatory markers, most importantly, troponin-I levels [11]. Endomyocardial biopsy was done in two patients and showed endo interstitial edema and neutrophils, along with mononuclear-cell infiltrates without giant cells. The left ventricular ejection fraction (LVEF) was severely reduced in four patients and was normal or mildly reduced in the rest of the study group. Cardiac magnetic resonance imaging (CMRI), a non-invasive magnetic resonance imaging method to assess the cardiovascular system, was done in 48 patients and showed at least one positive T1-based sequence and at least one positive T2-based sequence along with late gadolinium enhancement (LGE). The duration of the hospital stay was short, and there was normalization of echocardiogram and electrocardiogram (ECG) changes, along with normalization of inflammatory markers [11].

In a literature review by Nassar et al., 17 patients were considered and tracked, and their ECG and biochemical reports were retrieved. ECG showed T-wave inversion in two patients and ST-segment elevation in 12 patients. Cardiac troponin-I was elevated, along with a preserved ejection fraction, except in one patient who developed apical hypokinesia. CMRI showed mild myocardial edema and LGE in the subepicardial regions. Via this study, Nassar et al. were able to say that myocarditis was a probable side effect of COVID-19 vaccinations [12].

Kim et al. identified seven patients diagnosed with acute myocarditis and included four of them in their study who had recently received the mRNA COVID-19 vaccination. Three patients were young males, whereas one was a 70-year-old female, with two patients having received the Moderna vaccine and two patients having received the Pfizer-BioNTech vaccine one to five days before hospitalization. The four patients presented with common post-vaccination symptoms, including injection site discomfort, fever with chills, myalgia, fatigue, and cardio-centric symptoms like chest pain, palpitations, syncope, and shortness of breath, with all patients having no history of COVID-19 infection. The ECG showed diffuse ST-segment elevation and PR depression. Cardiac troponin-I levels were increased in all patients, with a few of them showing an increase in C-reactive protein (CRP), erythrocyte sedimentation rate (ESR), and pro-brain natriuretic peptide (pro-BNP) levels. Ejection fraction ranged between 40% and 59%, and regional wall motion abnormalities were seen in all patients on echocardiography. CMRI performed on all patients showed the presence of LGE with elevated native T1 in the region of LGE. Patients were treated with nonsteroidal anti-inflammatory drugs (NSAIDs), colchicine, and corticosteroids. The hospital stay lasted about two to four days and was uneventful [13].

Montgomery et al. presented a retrospective case series using members of the US military to establish a connection between COVID-19 vaccination and myocarditis. Overall, 2,810,000 doses were administered [14]. The study included 23 physically fit military members (22 active members and one retiree) with no history of cardiac disease, who presented with acute onset chest pain 12-96 hours following immunization with the COVID-19 vaccine. Twenty patients presented after the second dose, whereas three patients with a prior history of COVID-19 infection presented after only one dose of the vaccine. Sixteen patients received the Moderna vaccine, and seven received the Pfizer-BioNTech vaccine. All patients showed 10 to 400 times the normal troponin levels. ECG changes were seen in 19 cases, which included ST-segment elevations, T-wave inversions, and nonspecific ST changes. An echocardiogram showed reduced left ventricular ejection fraction (LVEF) ranging from 40%-50%, and CMRI done in eight patients showed subepicardial late gadolinium enhancement and focal myocardial edema, which were consistent with findings in myocarditis [14].

Singh et al. report a case involving a 24-year-old Caucasian man who presented to the emergency department (ED) with complaints of chest pain and was diagnosed to have acute myocarditis, having been vaccinated three days before his visit to the ED. On the day of vaccination, he developed systemic symptoms like fever, myalgia, and headache, which resolved in 24 hours [15]. He gave a history of strenuous physical activity, including shoveling snow for around 30 minutes the night before his ED visit. On presentation, the patient's vitals were normal, and the workup revealed an increase in high-sensitivity troponin-I, C-reactive protein (CRP), and erythrocyte sedimentation rate (ESR) levels. ECG changes included ST-segment depression in lead three, with an echocardiogram showing an ejection fraction of 55% and cardiac catheterization showing normal coronary arteries. CMRI showed linear subepicardial enhancement, consistent with myocarditis. His hospital stay of four days was uneventful, and he was discharged in stable condition. A follow-up after six weeks showed he was in stable condition and was back at work [15].

Clinical findings in adolescents

Jain et al. included 63 patients between the ages of 12 and 20 in their study [16]. Thirty-one patients were between 12 and 15 years old, and 58 were male, with 59 patients (94%) having received the Pfizer-BioNTech vaccine and four (6%) having received the Moderna vaccine. One patient, who did not have a previous known COVID-19 infection, presented after the first dose of vaccination, as compared to the others, who presented after the second dose. All patients showed elevated serum troponin levels. Most cases also had elevated levels of CRP, ESR, and pro-BNP. Abnormal ECG findings in 44 patients showed diffuse ST-segment elevation and/or T-wave inversion. Echocardiography showed a decreased left ventricular ejection fraction of 45%-54% in nine patients. Fifty-six patients underwent CMRI, with 51 of them doing so within a week. CMRI showed myocardial edema in 50 patients, and 49 patients showed late gadolinium enhancement (LGE) in the subepicardial region in a T2-based sequence [16].

In a study by Chelala, the clinical history, biochemical reports like elevated CRP and troponin levels, decreased ejection fraction on echocardiography, and cardiac MRI showing LGE were consistent with findings of myocarditis in five male patients aged 16 to 19 years. The mean length of hospital stay was 4.8 days, and all patients improved significantly and were discharged in stable conditions [17].

Dionne et al. reported a case series of 15 adolescents with a median age of 15 years who were admitted for the management of symptoms that developed between one and six days after receiving the Pfizer vaccine. Fourteen patients were male. All 15 patients had chest pain, 10 had a fever, eight had myalgia, and six had a headache [18]. Laboratory investigations showed elevated troponin-I levels. Echocardiograms in three patients showed global left ventricular systolic dysfunction; two patients showed systolic dysfunction with abnormal diastolic function indices; one patient showed diastolic dysfunction. More than 50% of patients had diffuse ST-segment elevation on their ECG. The most frequent finding on CMRI was late gadolinium enhancement found in the inferolateral and anterolateral regions. Intravenous immunoglobulins and methylprednisolone were used as a treatment in seven patients, with the mean hospital stay for all patients being two days and none requiring intensive care unit admissions. On follow-up, all patients appeared to be stable [18].

Management of COVID-19 vaccine-associated myocarditis

Oster et al. described 1991 reports sent to the Vaccine Adverse Effect Reporting System (VAERS), which occurred after at least the first dose of the mRNA vaccine. Of the 1626 patients that met the definition of probable or confirmed myocarditis, 676 cases had data available regarding the course of treatment. It was observed that 87% had received NSAIDS, 12% had received intravenous immunoglobulins, and 12% had received glucocorticoids. Twelve cases of myocarditis were put on vasoactive drugs, whereas two cases underwent intubation or mechanical ventilation. Besides these, anti-diuretics, anticoagulant therapy, antiarrhythmic drugs, and low- or high-flow nasal cannula oxygen support were also used. Follow-up after discharge revealed they were all doing well with the resolution of the presenting symptoms [19].

Management of myocarditis following the COVID-19 vaccine generally involved rest, NSAIDs, colchicine, steroids like methylprednisolone, and intravenous immunoglobulins [20]. Steroids must only be used until the resolution of symptoms or till the left ventricular functions have normalized [6]. Additionally, cardioprotective drugs like beta-blockers, angiotensin-converting enzyme inhibitors, and diuretics are also used. Usually, following a diagnosis of myocarditis, patients are asked to refrain from strenuous activity, physical stress, or any kind of activity that may bring about cardiovascular stress [20,21].

Myocarditis following vaccination in patients with a prior history of myocarditis

In a cohort study, Shahid et al. reported 34 patients with a history of myocarditis who had received at least two doses of the COVID-19 mRNA vaccine. The majority of the study group was male and white, with a median age of 41 years. Out of 34 patients, 26 had also received a third dose of the mRNA vaccine, with the majority having received the Pfizer vaccine. All 34 patients either had an in-person encounter or a phone encounter with their physician, with the median period of this encounter being nine months after their second dose. They reported that out of 34 patients, only one had a recurrence of myocarditis after the vaccination [22]. He was a white male who had the initial diagnosis at age 20 and had a recurrence at the age of 27 after receiving the second dose of the Moderna vaccine. He presented with chest pain two days after the vaccination and had elevated cardiac troponin levels (peak troponin 0.268 ng/ml) and sub-epicardial delayed enhancement on CMRI involving the basal inferolateral and inferior wall and distal lateral segment [22]. The distal lateral segment on CMRI seemed to have recently developed when compared to the CMRI report at the time of the first myocarditis diagnosis. After a hospital stay of two days, during which he did not require cardio-supportive treatment, he was discharged in a stable condition and was also stable at the post-discharge follow-up six weeks later. Shahid et al. concluded that the risk of developing recurrent myocarditis after vaccination was low. However, more studies need to be conducted with a bigger study group in order to arrive at a concrete conclusion [22].

Myocarditis following the third dose of vaccination

We found a study for vaccine-associated myocarditis occurring after the third dose, conducted by Simone et al. [23]. They included 3,076,660 individuals who had received at least one dose of the COVID-19 mRNA vaccine, of whom 2,916,739 received at least two doses and 1,146,254 received three doses. It was found that within 21 days of vaccination, there were nine cases of myocarditis after the third dose vaccination, with the incidence rate ratio being 2.61 (1.13-5.29) for the third dose. Eight of the nine patients were men, and five were aged between 18 and 40 years. They were treated conservatively, and on follow-up, they were stable with a resolution of symptoms (except for one patient who died of cancer). This study showed that there may also be a connection between the development of myocarditis and the third dose of vaccination [23].

Another case report by Brage et al. described a 62-year-old woman diagnosed with stage four lung adenocarcinoma affecting both lungs and lymph nodes who was treated with osimertinib [24]. She presented with fever and hypotension and showed tachycardia and skin pallor. On further history-taking, she revealed that she had been administered the third dose of the Moderna vaccine the previous day. The ECG revealed sinus tachycardia, and laboratory tests revealed elevated troponin I levels. A transthoracic echocardiogram revealed a dilated left ventricle with severely depressed LVEF, diffuse hypokinesia, and significant pulmonary congestion, suggestive of fulminant myocarditis with severe left ventricular dysfunction in cardiogenic shock. She was started on cloxacillin for 15 days and discharged with a course on corticosteroid therapy, colchicine, and antibiotics. Other possible causes of myocarditis were excluded after thorough studies, and literature on the drug osimertinib was also studied to discard it as a possible cause. Osimertinib was administered for around two years, and after a dose titration, the patient showed good tolerance towards it. This led them to conclude that the Moderna vaccine administered the previous day was the most probable cause for the sudden onset of symptoms [24].

Pathophysiology of myocarditis associated with COVID-19 vaccination

The pathophysiology of myocarditis is not fully understood, but it may be due to a direct effect on the cardiomyocytes or to an immune response stimulated by the vaccine [6]. The antibody response the host’s body has is due to the fact that the mRNA vaccinations produce viral spike proteins in the body that resemble myocardial proteins. This molecular mimicry causes myocardial injury or inflammation [6,16]. In some patients who have a genetic predisposition, there may be activation of the immunological memory cells, which leads to a systemic inflammatory response, causing the body to develop myocarditis [21].

Alternatively, the mRNA vaccine encodes for the viral spike protein, which may bind to the myocardial type 2 angiotensin-converting enzyme (ACE2) receptors present in the cardiomyocytes [16,17]. This combination of proteins causes an immune response in the host’s body [16]. The most likely mechanism may be due to the molecular mimicry between spike proteins present on the SARS-CoV-2 viral particles from the vaccine and antigens present in the body. There may be a cross-reaction between the antibodies developed against the SARS-CoV-2 spike proteins and structurally similar human protein sequences, for instance, proteins present in the myocytes [7].

**Figure 1:**
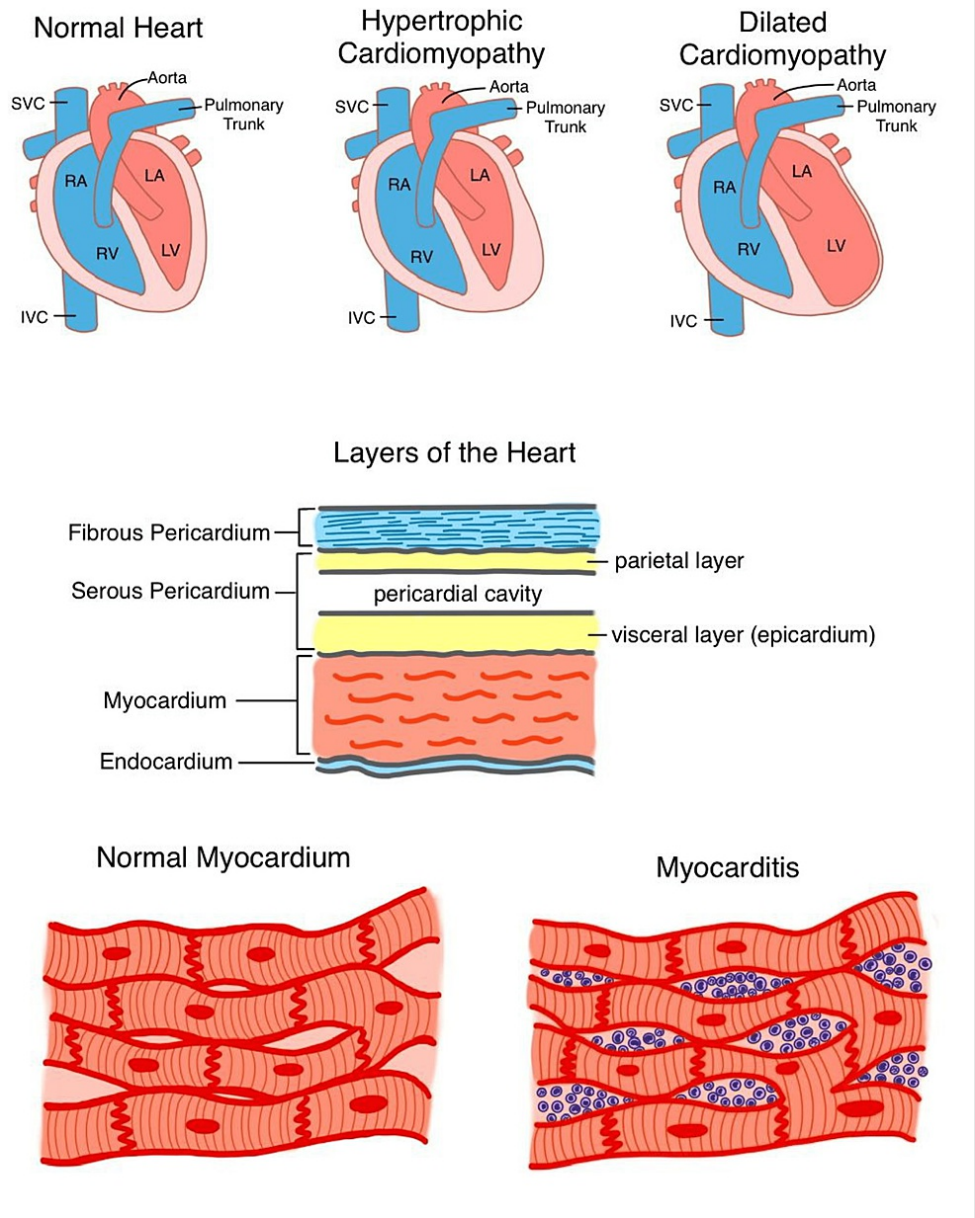
Normal myocardium versus myocarditis SVC: superior vena cava; IVC: inferior vena cava; RA: right atrium; LA: left atrium; RV: right ventricle; LV: left ventricle This image is an original illustration by the second author, Tejasvi Kashyap.

Figure 1 shows the comparison between normal myocardium and myocarditis, along with hypertrophic and dilated cardiomyopathy and layers of the heart.

## Conclusions

Through this review, we aimed to establish an association between myocarditis and the COVID-19 vaccination. We found that there is increased reporting of cases of myocarditis in proximity to the period of vaccination. The most commonly affected population was the younger age group, between 16 and 30 years, with males being affected more than females. However, post-vaccination management of COVID-19 patients was found to be mostly conservative in the form of oral medications, though more research will likely help to better compartmentalize the type of treatment for different manifestations of various vaccine strains. Vaccinations that cause myocarditis are also a rising concern among the adolescent population. For a better study of patients who are treated for myocarditis following COVID-19 vaccination, better methods for follow-up and post-discharge CMR must be initiated. High-quality, large-scale, randomized trials and evidence on pathophysiology may be needed in the future to strongly connect and study myocarditis post-vaccination in order to set proper guidelines regarding treatment strategies for the same.
